# Efficacy and safety of Zuojin Pill for chronic gastritis: Protocol for a systematic review of randomized controlled trials

**DOI:** 10.1097/MD.0000000000021248

**Published:** 2020-07-17

**Authors:** Jianglong Shi, Liyun Liu, Jian Li, Xiaoju Ma, Hairong Qiu, Tao Shen

**Affiliations:** a School of Basic Medicine Sciences, Chengdu University of Traditional Chinese Medicine , Chengdu, Sichuan; b Department of Sports Medicine, Shanghai University of Sport, Shanghai, China.

**Keywords:** chronic gastritis, protocol, randomized controlled trials, systematic review, traditional Chinese medicine, Zuojin Pill

## Abstract

**Background:**

Chronic gastritis (CG), as the highest incidence of gastrointestinal diseases, has been gradually increasing around globally. With the obvious disadvantages of standard treatment, more and more people ask the traditional Chinese medicine for help in the treatment of CG. As a traditional Chinese medicine compound, Zuojin Pill (ZJP) has a long history of clinical application in the treatment of digestive system diseases. Whereas, neither systematic nor meta-analysis of randomized controlled trials explain the efficacy and safety of ZJP in treating CG. Thus, we provide a protocol to evaluate the efficacy and safety of ZJP for CG.

**Methods:**

From the beginning to December 2019, the following electronic databases will be searched for studies in English or Chinese: the Cochrane Library, Embase, PubMed, Web of Science, the Chinese National Knowledge Infrastructure, the Chinese Biomedical Literature Database, the Chinese Scientific Journal Database, and the Wanfang Database. Clinical efficiency, helicobacter pylori infection clearance rate, quality of life and symptom scores will be measured as primary outcomes. Meta-analysis will be performed using the Stata 15.

**Outcomes:**

This study will provide the current evidence of CG treated with ZJP from the several aspects including clinical efficiency, helicobacter pylori infection clearance rate, quality of life, symptom scores, the 1-year recurrent rate, efficacy under endoscopy and number of reported adverse events associated with the use of ZJP.

**Conclusion:**

The outcomes of this review will be served as a proof to evaluate if ZJP is effective in the treatment of CG.

**PROSPERO registration number:**

PROSPERO CRD42020155036.

## 1 Introduction

Chronic gastritis (CG) is a chronic inflammation of gastric mucosa, caused by various reasons,^[1]^ usually along with clinical manifestations such as stomachache, distension, loss of appetite, belching, pantothenic acid, nausea, vomiting and other symptoms. According to the Sydney System, CG, with the pathological features including inflammation, atrophy, and intestinal metaplasia, is divided into non-atrophic, atrophic and specific.^[2,3]^ The incidence of CG in China has exceeded 60%.^[4,5]^ Among many pathogenic factors, helicobacter pylori (HP) infection is the main cause.^[6,7]^ It has been confirmed that Hp is closely related to diseases of the upper gastrointestinal tract.^[8,9]^ With the high infection rate of Hp in China, there is an urgent need for a more timely and effective treatment of CG.^[4]^ Mucosa protectants, gastric acid inhibitors, spasmodic agents, and motility enhancers are commonly used in the treatment of CG. For HP infection, clinical triple therapy is commonly used.^[10]^ However, because of the poor compliance of patients,^[11]^ emerging resistant HP strains^[9,12–14]^ and adverse drug reactions,^[15–17]^ the clinical eradication rate of HP has decreased to an unacceptable low level,^[18]^ which lead to recurrence frequently.^[19,20]^ The long course of the disease needs to take medicine for a long time, causing great psychological pressure^[21,22]^ to the patient and increasing substantial healthcare costs, which cause significant reductions in patients’ quality of life.^[23]^ Nevertheless, conventional treatment has been proved to represent either limited efficacy or often recur after the withdrawal of drugs.^[24]^ While new drugs have improved health, traditional therapies have often failed to treat CG gratifyingly. By reasons of its good curative effect and few adverse reactions and long history of treating chronic diseases,^[25]^ traditional Chinese medicine has been accepted by more and more people all over the world.

Zuojin Pill (ZJP)^[26]^ is a traditional Chinese medical formula developed by Danxi Zhu, an outstanding physician of the 4 medical schools in the Jin and Yuan Dynasty. This classical prescription has been used by numerous herbalists for CG for hundreds of years. ZJP consists of the 2 Chinese herbs, namely *Coptis chinensis Franch* (Huang Lian) and *Tetradium ruticarpum* (Wu Zhu Yu), with a specific dose ratio of 6:1. It helps to clear of liver heat, down bear the reversed Qi, stop vomiting, relieve abdominal stuffiness and fullness.

*Coptis chinensis Franch*, the herb with bitter flavor and cold property, enters into the channels of heart, liver, stomach and large intestine, and functions on clearing heat, drying dampness, purging fire and detoxification. *Tetradium ruticarpum*, the herb with spicy flavor and warm property, enters into the channels of liver, spleen, stomach and kidney, and functions on dispersing cold and relieving pain, down bear the reversed Qi, stop vomiting, and strengthen yang to relieve diarrhea. The employment of *Coptis chinensis Franch* and *Tetradium ruticarpumcontrary* but complementary, has a good curative effect on the symptoms caused by gastrointestinal diseases such as poor appetite, abdominal pain, belching, acid regurgitation, nausea, vomiting, and so on.

As could be observed from pharmacologic studies, ZJP has obvious inhibitory effects on gastric acid secretion, gastric ulcer, bacteriostasis, anti-inflammatory and analgesia. Meanwhile, recent evidence presents that ZJP can treat gastrointestinal disease and had remarkable anti-inflammatory effects. Recently, with the publication of numerous trials on ZJP for CG, they have proved that ZJP has good clinical effect. There is an pressing need for a systematic review to support the effectiveness and safety of ZJP in treating CG. Therefore, the purpose of the study is to systematically review current available articles to assess the efficacy and safety of the ZJP treatment in patients of CG.

## 2 Method

### 2.1 Study registration

This protocol for system review has been registered in the PROSPERO database (registration number: CRD42020155036). The review reporting will be developed in compliance with the guidelines of the Preferred Reporting Items for Systematic review and Meta-Analysis Protocols and conducted with The Cochrane Handbook for Systematic Reviews of Interventions.

### 2.2 Inclusion criteria

#### 2.2.1 Study characteristics

Only randomized controlled trials (RCTs) will be included instead of case reports, narrative reviews, systematic reviews, or cross-over trials. Studies lacking complete and accurate data and neither published in Chinese or English will be excluded. Restrictions will not be imposed on publication status.

#### 2.2.2 Participants

Participants meeting the diagnostic standard of the second national consensus on CG will be involved irrespective of their age, sex, or ethnicity. Patients with other complicating diseases will not be included.

#### 2.2.3 Intervention

In treatment group, ZJP or modified ZJP will be the sole treatment for patients, while routine western medicines will be used alone in control group. ZJP consists of *Coptis chinensis Franch* (huang lian) and *Tetradium ruticarpum* (wu zhu yu). Modified ZJP will also be included as long as it contains the 2 herbs and increases not exceeding 10 herbs. All the formulas involved should keep to the principles of Monarch, Minister, Assistant, and Guide in traditional Chinese medicine prescription. There is no restriction on dosage form or mode of administration. The control group: basic treatment, including routine medication and health education, was not allowed to accept any form of ZJP treatment during the study period.

#### 2.2.4 Outcomes

The following primary outcomes will be measured: clinical efficiency, HP infection clearance rate, quality of life and symptom scores. The secondary outcomes will be the 1-year recurrent rate, efficacy under endoscopy and number of reported adverse events associated with the use of ZJP.

### 2.3 Search strategy

#### 2.3.1 Electronic searches

A detailed search will be conducted by using the following electronic databases from the beginning to December 2019: the Cochrane Library, Embase, PubMed, Web of Science, the Chinese National Knowledge Infrastructure, the Chinese Biomedical Literature Database, the Chinese Scientific Journal Database, and the Wanfang Database. The keywords include “Zuojin pill”, “chronic gastritis”, etc. The search strategy for PubMed is summarized in Table 1. Relevant data will also be searched through other sources: hand searching, conference proceeding, International Clinical Trials Registry Platform, and Chinese Clinical Trial Registry.

**Table 1:**
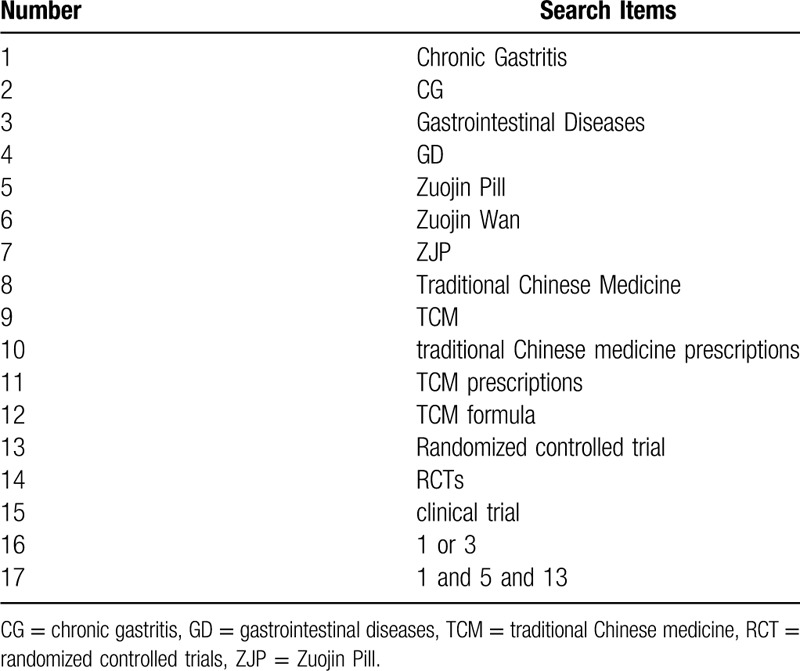
Search strategy for PubMed.

### 2.4 Data collection and analysis

#### 2.4.1 Selection of studies

**Figure 1:**
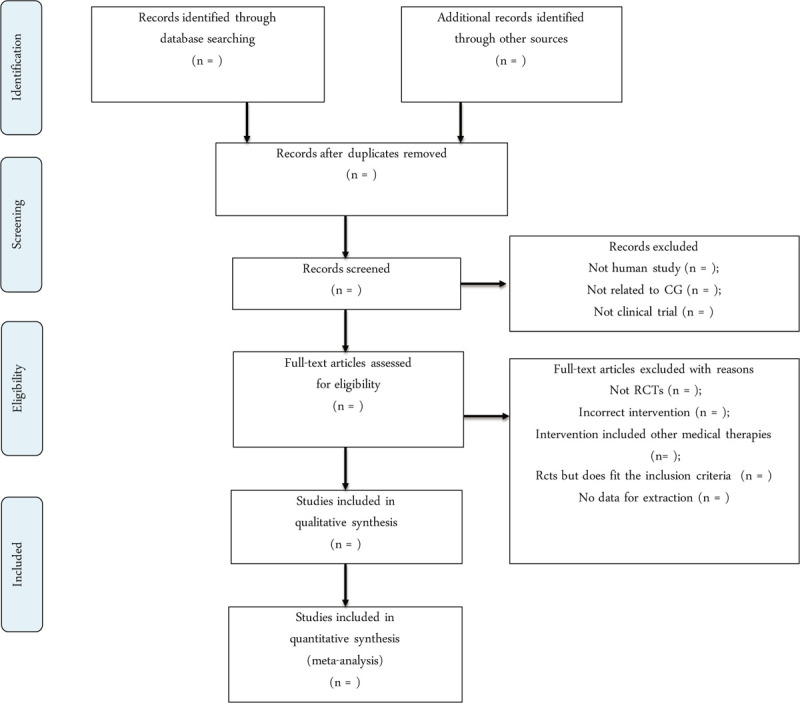
Preferred Reporting Items for Systematic Reviews and Meta-Analyses flow chart of study selection process.

The search results from all database will be combined and the duplicates will be removed by the EndNote X9. According to the inclusion criteria, 2 investigators (Jianglong Shi and Liyun Liu) will select potentially eligible studies by assessing the titles and abstracts independently. Subsequently, 2 investigators will read over the full texts of the included studies and communicate with each other to make a final selection. Some studies will be removed because of below reasons: non-RCTs or quasi-RCTs and animal study, journal or conference proceedings with no associated full-text article and no data for extraction. Any divergence will be dealt with by the discussion with a 3rd reviewer (Tao Shen). The whole selection process will be presented in a preferred reporting items for systematic reviews and meta-analyses flow diagram (Fig. 1).

#### 2.4.2 Data extraction

The data extraction will be performed by 2 investigators (Jian Li and Xiaoju Ma) through a predefined form, including 4 parts: basic information, characteristics of trial subjects, intervention measures, and results of the studies. Identically, the 3rd reviewer (Tao Shen) will make a final decision in case of discrepancy. If some information is insufficient, we will make an attempt to contact the authors of the original trial. Supposing that the author fails to respond, the study will be discarded and only the available data will be analyzed. The influence caused by the missing data on the meta-analysis results will be taken into consideration.

#### 2.4.3 Quality evaluation on methodology

Taking the criterion in the Cochrane Handbook for Systematic Review of Interventions V.5.2.0 (renovated June, 2017), risk of bias will be classified into 3 categories (low, unclear and high) independently by 2 investigators. Overall, the quality assessment will be based on the 7 domains: random sequence, blinding of the participants and personnel, allocation concealment, blinding of outcomes, selective reporting, completeness of outcome data, and other bias. In case of discrepancy, consensus will be reached by a collective discussion.

#### 2.4.4 Statistic analysis

Meta-analysis will be carried through using the Stata 15. The continuous outcome data will be expressed as mean differences, while the dichotomous outcomes will be analyzed by using risk ratios with 95% confidence interval using fixed or random-effect models.

#### 2.4.5 Assessment of heterogeneity

Statistical heterogeneity among the studies will be assessed by *I*^2^ and Chi-squared statistics. Heterogeneity will be considered to be considerable if *I*^2^ ranges from 50% to 100%, for which we will analyze data using a random-effect model. If the tests for heterogeneity have no significant meaning (*I*^2^ ≤50%), the fixed effect model will be used.

#### 2.4.6 Assessment of reporting bias

Funnel plots will be conducted to evaluate reporting bias. If potential reporting basis detected, Begg and Egger test will serve to evaluate the symmetry of the funnel plot and perceive publication bias.

#### 2.4.7 Sensitivity analysis

Sensitivity analysis will be conducted to identify the robustness of the result and detect whether there are any exceptional studies bringing about an evident heterogeneity. Then the particular study will be scrutinized to find the reasons.

#### 2.4.8 Subgroup analysis

Subgroup analyses will be performed to explore the source of heterogeneity according to the following items: the duration or severity of CG, children or adults, the original or modified of ZJP, and the duration or dosage of herbal medicine treatment.

#### 2.4.9 Quality of evidence

The dependability of proof will be appraised by the Grading of Recommendations Assessment, Development, and Evaluation. The following factors will be taken into consideration: limitations in the design, unaccounted heterogeneity, discrepancy, indirectness of evidence, hidden error, and selective publication. Evidence quality will be rated as high, moderate, low, or very low.

#### 2.4.10 Ethics and dissemination

It is our aim that this review is going to be shed in peer-reviewed journals. Private information from individuals will not be involved in the review, so there is no need for informed consent form. Ethical approval is also unnecessary because this study is not a clinical trial. Neither patients nor public got involved.

## 3 Discussion

CG is a chronic inflammatory digestive tract disease, which is a chronic process of gastric mucosa injury and repair. The causes of CG include HP infection, autoimmunity, bile and pancreatic juice flowing into the stomach from the duodenum, alcoholism, the use of NSAIDs and the intake of stimulating foods.^[7]^ Repeated injury of gastric mucosa may further result in atrophy of gastric mucosa leading to cellular atypia and disorder of glandular structure, namely, precancerous lesions of gastric cancer. For those with a longer duration of CG, standard treatment can only control part of the symptoms. Due to recurrent attacks and great economic pressure, it is easy to be anxiety and tension of patients, which reducing the quality of life of patients. ZJP, a classical traditional Chinese medicine formula, is diffusely used for patients with CG. It has more advantages than single receptor chemicals in treating CG with multicomponent and multitarget therapy. These analogous formulas all have commonness in protecting gastric mucosa and superiorities of evidence-based derivation in compliance with multilevel effect evaluation, whose effect pathway was involved in Cell structure protection, anti-inflammation, anti-oxidation, immune regulation. However, a systematic review of ZJP in treating CG has not yet been published. This systematic review will be the first to provide a summary of the current state of proof concerning the effectiveness and safety of ZJP in treating CG. This evaluation will be useful for doctors and patients with CG.

## Author contributions

Jianglong Shi and Liyun Liu are joint first authors.

Tao Shen obtained funding.

Jianglong Shi and Jian Li designed the study.

Jianglong Shi drafted the manuscript.

Xiaoju Ma and Hairong Qiu contributed to critical revision of the manuscript for important intellectual content and approved the final version of the manuscript.

All authors have read and approved the final manuscript.

Tao Shen is the study guarantor.

**Funding acquisition:** Tao Shen.

**Investigation:** Xiaoju Ma.

**Methodology:** Jian Li.

**Resources:** Hairong Qiu.

**Supervision:** Tao Shen.

**Writing – review & editing:** Liyun Liu.
